# Early-Life Iron Exposure Influences Long-Term Gut Microbiota Recovery After Intestinal Dysbiosis

**DOI:** 10.3390/microorganisms14051105

**Published:** 2026-05-13

**Authors:** Thibault Maumy, Claire McCartney, Ayodeji Samuel Ajayi, Claire Gerkins, Gabriela Fragoso, Annie Calvé, Manuela M. Santos

**Affiliations:** 1 Nutrition and Microbiome Laboratory, Institut du Cancer de Montréal, Centre de Recherche du Centre Hospitalier de l’Université de Montréal (CRCHUM), Montréal, QC H2X 0A9, Canada; thibault.maumy@umontreal.ca (T.M.);; 2 Department of Medicine, Faculty of Medicine, Université de Montréal, Montréal, QC H3T 1J4, Canada

**Keywords:** iron, early life, microbiota, dysbiosis, microbiota recovery

## Abstract

Host–microbiota interactions during the neonatal window of opportunity have gained significant interest as factors influencing long-term health. Factors such as nutrient availability may shape the microbial community, and iron is no exception to this rule. Although the use of iron supplementation is widespread during infancy, this micronutrient is known to have profound effects on gut microbiota. This study aimed to determine how early-life iron supplementation shapes gut microbiota composition and whether it influences recovery from gut dysbiosis later in life. Three-week-old female C57BL/6 mice were fed an iron-excess diet for five weeks during the critical period of microbiota establishment. After a two-week washout period to normalize luminal iron content, dysbiosis was induced using either dextran sulfate sodium-induced acute colitis or antibiotic treatment. Mice were then allowed an 8-week recovery period. Gut microbiota composition was longitudinally analyzed by 16S rRNA gene sequencing of fecal samples. Early-life iron supplementation induced durable alterations in gut microbiota composition, with differences persisting even after luminal iron normalization (β-diversity, PERMANOVA *p* < 0.01). At the endpoint, mice exposed to an iron-sufficient diet remained significantly more distant from their baseline compared to the excess iron group in both the dextran sulfate sodium (33% greater distance) and antibiotic (41% greater distance) models (both *p* < 0.05). Notably, this pattern was not observed when supplementation occurred in adulthood. In the dextran sulfate sodium model, mice that received an iron-sufficient diet during early life showed an expansion of the probiotic *Ligilactobacillus murinus*, which positively correlated with fecal succinate levels. Conversely, in the antibiotic-induced model, early-life exposure to an iron-sufficient diet was associated with a more pronounced dysbiosis characterized by a nearly two-fold-greater loss in α-diversity compared to 500 ppm mice (∆Shannon: 1.98 ± 0.22 vs. 1.02 ± 0.25, *p* < 0.01). These findings suggest that early-life iron supplementation influences long-term host–microbiota interactions and recovery from gut dysbiosis.

## 1. Introduction

There is mounting evidence that the gut microbiome plays a significant role in shaping the health of its host [1], as this consortium of microorganisms residing in the mammalian gut confers numerous benefits and interacts closely with the host immune system [2]. Multi-omics approaches integrating metagenomics and metabolomics have shown that microbial-derived metabolites—such as short-chain fatty acids (SCFAs), bile acids and tryptophan derivatives—act as key mediators in these interactions [3,4]. Beyond local intestinal effects, the gut microbiota also exerts systemic influences through axes such as the gut–brain and gut–muscle axes [5,6], modulating neurological function, metabolism, and host physiology. In this complex interplay, balance is key: disruption to the gut microbiota, known as dysbiosis, has been linked to several diseases [7]. Recovery from dysbiosis is essential to restore the benefits and protection provided by a healthy gut microbiota. Accordingly, gut microbiome-targeted interventions are increasingly popular to improve disease outcomes [8,9].

In humans, the gut microbiota is established from birth until approximately three years of age, after which it remains relatively stable throughout adulthood [10]. This development phase is often referred to as the neonatal window of opportunity, reflecting the dynamic interplay between microbiota colonization and immune system development [11]. Multiple environmental and nutritional factors modulate early-life gut microbiota establishment, including mode of delivery, early-life feeding practices (breast milk versus formula), antibiotic exposure, diet, and micronutrient availability, and have been linked to long-term health outcomes and disease susceptibility [12].

Iron is one such factor with the potential to strongly influence early-life gut microbiota establishment. As an essential micronutrient for both the host [13] and microorganisms [14], iron availability within the gastrointestinal tract may play a critical role in shaping the gut microbial community.

At the cellular level, iron supports growth by acting as a cofactor in key processes such as respiration, DNA synthesis, and central metabolism. However, when present in excess, free iron can become deleterious by catalyzing the formation of reactive oxygen species through the Fenton reaction, leading to oxidative stress that damages both microbial cells and host tissues [15]. As a result, iron exerts a strong selective pressure in the gut ecosystem because it is an essential nutrient required for bacterial metabolism, yet it can also be toxic when present in excess. Consequently, bacteria must not only efficiently acquire iron but also possess mechanisms to protect themselves against iron-induced toxicity [16]. In mammals, iron homeostasis is tightly regulated at the level of intestinal absorption [13], as there is no dedicated excretory pathway for excess iron [17]. As a result, dietary iron supplementation has been shown to directly alter microbial composition and diversity in both mice and humans [18,19,20].

On the other hand, iron deficiency is a major health issue and is particularly detrimental during infancy, as it can impair development. Consequently, oral iron supplementation is widely recommended for infants and young children [21], commonly in the form of ferrous sulfate. Beyond therapeutic use, iron is also broadly available over the counter and is routinely included in fortified infant formulas [21]. Although breastfeeding is globally recommended, infant formula use remains widespread [22]. Notably, current formulations often contain iron concentrations several-fold higher than the levels considered sufficient for healthy-term infants [23]. In addition to these dietary sources, a Canadian survey reported that 8.8–15.7% of children aged 1–3 years receive supplemental iron [24].

In individuals without iron deficiency, however, dietary iron supplementation may increase the amount of unabsorbed iron reaching the colon, potentially altering gut microbial composition. Several studies have demonstrated that excess luminal iron during early life can modify the gut microbiota of infants in countries with a lower socio-economic index [25,26], but there is no data regarding early-life supplementation in countries with a high socio-economic index, where iron needs in children and young infants may be different and where supplementation is still common [27].

Nevertheless, it remains unclear whether early-life iron overload durably conditions the microbiota in ways that influence its resilience or recovery from dysbiosis later in adulthood. We hypothesized that early-life exposure to dietary iron excess influences the establishment of gut microbiota, leading to compositional changes that modulate the trajectory and efficiency of microbiota recovery following dysbiosis in adulthood.

## 2. Materials and Methods

### 2.1. Animal Experiments

All animal procedures were conducted in accordance with the Canadian Council on Animal Care and approved by the Institutional Animal Care Committee of the CHUM Research Center (2024-11660).

A colony of wild-type C57BL/6 mice (Charles River Laboratories, Saint-Constant, QC, Canada) was established at the animal facility of the CHUM Research Center to obtain the 96 juvenile female mice used in this study. Breeding pairs were set up simultaneously, with one male and two females per cage, ensuring that parental lines were unrelated. At the start of the experiment, three-week old mice were housed post-weaning in pairs from different litters to limit potential litter or genotype effects. Two independent experiments were conducted using identical designs, differing only in the dysbiosis model used (dextran sulfate sodium (DSS) or antibiotics).

In each experiment, 48 mice were randomly assigned to two dietary groups (n = 24/group): an iron-sufficient diet (50 parts per million (ppm) iron, FeSO_4_; Teklad™ Diet TD.120515, Inotiv, West Lafayette, IN, USA) consistent with adequate iron levels, or an iron-excess diet (500 ppm iron, FeSO_4_; Teklad™ Diet TD.120517, Inotiv) representing an ~10-fold increase over basal dietary iron exposure [23,28,29]. After 35 days, all mice were transitioned to an iron-sufficient diet (50 ppm) for two weeks, a period sufficient to normalize luminal iron levels, given that whole-gut transit time in mice is typically on the order of a few hours [30]. Moreover, colonic epithelial cells undergo rapid turnover, with complete renewal occurring within approximately 5–7 days. Because iron-loaded enterocytes are continuously shed into the intestinal lumen through desquamation, this additional period further limits the persistence of excess luminal iron [31]. This washout phase ensured that subsequent responses to dysbiosis-inducing treatments were attributable to early-life dietary iron exposure rather than to acute differences in colonic iron levels. Mice were further divided into 4 groups (n = 12/group) and given 0.75% DSS (TdB Labs, product DB001, Uppsala, Sweden) in drinking water for 5 days to induce colitis or a cocktail of metronidazole (500 mg/L) and ciprofloxacin (200 mg/L) in drinking water for 7 days. Sterile drinking water was administered to untreated groups (50 and 500 ppm iron; n = 12/group). This was followed by an eight-week recovery period, during which all mice remained on the iron-sufficient diet. Experiments following the same design were conducted with 80 female adult mice (at least 8 weeks of age; n = 12 per group) to assess if the effects observed were specific to early life.

During DSS exposure, mice were monitored daily and disease severity was evaluated using a disease activity index (DAI) score based on three parameters: stool consistency, presence of rectal bleeding, and body weight change [32]. Outcome assessment was performed blinded to group allocation. Exclusion criteria included reaching humane endpoints (>20% weight loss, severe lethargy, dehydration or persistent bleeding). Body weight variation was calculated relative to the first day of DSS administration. Each parameter was scored individually, and the scores were added to obtain the final DAI. Scoring criteria was as follows: stool consistency (normal = 0; loose = 2; diarrhea = 4); bleeding (none = 0; hemoccult positive = 2; rectal bleeding = 4), and body weight loss (0% = 0; 1–5% = 1; 5–10% = 2; 10–20% = 3; >20% = 4).

Coprophagy effects and cage-specific influences on gut microbiota composition were limited by exchanging litter between cages [33] within the same experimental group during the first seven weeks prior to DSS or antibiotic exposure.

To monitor gut microbiota composition, fecal samples were collected from mice at multiple timepoints. Timepoints for mice exposed during early life: at weaning (Day 0) and at the end of the treatment (respectively Day 54 and Day 56). Timepoints for mice exposed during early life and adulthood: at the end of the dietary exposure (Day 35), prior to DSS or antibiotic administration (Day 49), and at the end of the experiment (Day 112). Samples were placed in standard 1.5 mL microcentrifuge tubes, immediately snap-frozen in liquid nitrogen, and stored at −80 °C until further processing. As a subset of samples from early-life-exposed DSS-treated mice yielded no sequencing reads at Day 54, microbiota composition was assessed using stool samples collected the day prior (Day 53) for those animals.

A total of 176 mice were used across all experiments. In the early-life DSS experiment, one mouse in the 500 ppm DSS group was euthanized due to severe clinical signs consistent with acute colitis. Similarly, in the adult DSS experiment, one mouse from the 500 ppm DSS group met humane endpoint criteria and was euthanized. In the early-life antibiotic experiment, one mouse in the 500 ppm untreated group was euthanized due to an acute non-treatment-related condition characterized by severe limb inflammation and impaired mobility. These mice were excluded from all downstream analyses.

Sample size (n = 12 per group) was determined based on commonly used group sizes in murine DSS-colitis and microbiota studies and was considered sufficient to detect biologically meaningful differences while accounting for inter-individual variability and ethical constraints related to animal use.

### 2.2. DNA Extraction, Sequencing and 16S rRNA Data Analysis

Bacterial DNA was extracted using the DNeasy PowerSoil Pro Kit (47014, Qiagen, Germantown, MD, USA) according to the manufacturer’s instructions. 16S ribosomal RNA (rRNA) libraries were prepared and sequenced at the Genome Québec facility on the Illumina NextSeq platform (Illumina, Inc., San Diego, CA, USA), targeting the V5–V6 region of the 16S rRNA gene (P609D (S-D-Bact-0785-a-S-18) 5′-GGMTTAGATACCCBDGTA-3′ and P699R (S-*-Univ-1100-a-A-15) 5′-GGGTYKCGCTCGTTR-3′) and sequencing 2 × 300 bp paired-end Polymerase Chain Reaction (PCR) products [34].

Primer sequences were trimmed from raw forward and reverse demultiplexed 16S rRNA gene reads using Cutadapt (v4.8) [35]. DADA2 (v3.18) [36] implemented in R (v4.3.0) was then used to denoise reads, infer Amplicon Sequence Variants (ASVs), and remove chimeric sequences. ASVs were taxonomically annotated using DADA2’s assignTaxonomy and addSpecies functions with the SILVA 138.2 reference database [37]. For some ASVs lacking species-level annotation after DADA2 classification, manual annotation was performed using BLASTn (2.17.0) [38] searches against the NCBI rRNA/ITS database [39]. α-diversity metrics were calculated using the phyloseq package (v1.52.0) [40]. To improve detection of differentially abundant ASVs and reduce sparsity-driven noise, low-abundance features were filtered prior to downstream analyses. ASVs were excluded if they had fewer than 10 total counts across all samples or were present in fewer than 5% of samples. β-diversity was assessed using weighted UniFrac distances and visualized by Principal Coordinates Analysis (PCoA) implemented in phyloseq. ASV sequences were aligned, a distance matrix was computed using the DECIPHER package (v3.4.0) and a phylogenetic tree was constructed using the neighbor-joining method implemented in ape package (v5.8.1). Visualizations were created with ggplot2 package (v4.0.1) and stacked bar graphs were created using the StackbarExtended package (v1.0) [41].

### 2.3. Differential Abundance Testing

Differential abundance testing was performed using DESeq2 (v1.48.1) [42] with a Wald test (fitType = “local”) with false discovery rate (FDR) correction. For diet effects at Days 35 and 49, models included batch as a blocking factor (~diet + batch) to account for cohort-specific variation. Analyses of DSS- or antibiotic-treated groups were conducted on relevant sample subsets. Longitudinal changes were assessed using a paired design (~mouse_id + timepoint). At the endpoint, differential abundance was tested using a factorial model (~diet * treatment), including untreated controls to capture baseline variability and diet-dependent responses to dysbiosis. To specifically evaluate long-term effects of early-life iron exposure on recovery, downstream comparisons were restricted to treated groups (50 ppm vs. 500 ppm; DSS or antibiotics). For higher-level taxonomic analyses (phylum and family), ASVs lacking classification at the level of interest were excluded prior to testing to reduce multiple-testing burden and improve FDR correction.

### 2.4. PICRUSt2 Functional Profile Inference

MetaCyc [43] pathway abundances were inferred from ASV data using PICRUSt2 (v2.6.2) [44] with the updated SC database [45]. Briefly, ASVs were aligned to a reference phylogenetic tree, and gene family abundances were predicted based on the nearest sequenced taxon and collapsed into MetaCyc pathways. Pathways were retained if present in at least three samples to reduce the influence of low-prevalence and potentially spurious features. Differential abundance analysis at the endpoint was performed using the DESeq2 package (v1.48.1) and following the same principles as described in the previous section. A Z-score was calculated for each sample to represent relative pathway abundances in individual mice, while normalizing the high disparity between pathway abundance counts.

### 2.5. Quantitative Polymerase Chain Reaction for Bacterial Species

Quantitative PCR (qPCR) was used to validate the abundance of bacterial candidates identified by 16S rRNA analysis. Reactions were performed using PowerUp™ SYBR™ Green Master Mix (A25777, Applied Biosystems, Waltham, MA, USA) with primers based on previous publications or designed using Primer-BLAST (https://www.ncbi.nlm.nih.gov/tools/primer-blast/, accessed 20 February 2026) [46], detailed in Table A1. All qPCRs were run in duplicate to ensure reproducibility.

### 2.6. Stool Iron Measurements

Fecal iron levels were measured using a ferrozine-based colorimetric assay at 595 nm [47], using the QuantiChrom Iron Assay Kit (BioAssay Systems, Hayward, CA, USA) according to the manufacturer’s protocol.

### 2.7. Measurement of Fecal LCN2

Fecal lipocalin-2 (LCN2) was quantified using the Mouse Lipocalin-2/NGAL DuoSet ELISA kit (DY582, R&D Systems/Bio-Techne, R&D Systems, Minneapolis, MN, USA) following the manufacturer’s instructions.

### 2.8. Short-Chain Fatty Acid and Bile Acid Measurements

Short-chain fatty acids (SCFAs) and bile acids (BAs) were quantified by LC-MS/MS with isotope dilution using external calibrators at the metabolomics core facility of the CRCHUM. Stools of approximately 20 mg were homogenized manually in 50% aqueous acetonitrile (30 µL per mg sample) using a polypropylene pestle, vortexed, and centrifuged at 20,000× *g* for 15 min at 4 °C. Short-chain fatty acids were measured using a method modified from Han J et al. 2015 [48]. Briefly, 30 µL of supernatants, blanks and standards were transferred to glass tubes with 10 µL of a 50% aqueous acetonitrile solution containing deuterated internal standards (acetic acid-d4, propionic acid-d5, butyric acid-d2, isobutyric acid-d7, valeric acid-d2, isovaleric acid-d2, caproic acid-d3 (CDN Isotopes, Pointe-Claire, QC, Canada)). Carboxyl groups were derivatized using 3-nitrophenylhydrazine as published, in a final total volume of 80 µL. After dilution to 1mL in 10% aqueous acetonitrile, derivatized organic acids were separated by reversed-phase chromatography (Nexera X2, Shimadzu, Kyoto, Japan) using a C18 column (Poroshell 120 EC-C18, 2.1 × 75 mm, 2.7 µm, Agilent, Santa Clara, CA, USA), and detected by ESI-MS/MS in negative-ion mode (QTRAP 6500, SCIEX, Framingham, MA, USA). Bile acids were measured on the same homogenates, diluting supernatants in 7.5 volumes of 30% aqueous acetonitrile solution, acidified with 0.25% trifluoroacetic acid, and containing CA-d4, DCA-d4, CDCA-d4, GDCA-d4, GUDCA-d4, TDCA-d4 (CDN Isotopes, Pointe-Claire, QC, Canada), UDCA-d4, HDCA-d5, TCA-d5, TCDCA-d5, LCA-d4 (Toronto Research Chemicals, Vaughan, ON, Canada), α-MCA-d5, β-MCA-d5 and ω-MCA-d5 (Cambridge Isotope Laboratories, Andover, MA, USA). After centrifugation (20,000× *g*, 15 min at 4 °C), bile acids from the supernatants were separated by reversed-phase chromatography (Nexera X2, Shimadzu) using a C8 column (Poroshell 120 HPH-C8, 2.1 × 100 mm, 2.7 µm, Agilent) in a gradient elution performed in water with 0.1% formic acid and acetonitrile and 0.1% formic acid, at 40 °C. Analytes were detected by ESI-MS/MS in negative-ion mode (QTRAP 6500, SCIEX).

### 2.9. Statistical Analysis and Sample Exclusion

Statistical analyses were performed using R (v4.3.0). Tests were selected based on data distribution and experimental design, with repeated-measures approaches applied to longitudinal data where appropriate. A *p*-value of < 0.05 was considered statistically significant. Normality was assessed using the Shapiro–Wilk test, and homogeneity of variances was evaluated using Levene’s test from the car package (v3.1.5).

Comparisons between two groups were performed using Student’s *t*-test or Welch’s *t*-test for normally distributed data (depending on variance homogeneity), and the Mann–Whitney U test for non-normally distributed data. Paired comparisons between two timepoints were assessed using paired *t*-tests.

β-diversity analyses were conducted using permutational multivariate analysis of variance (PERMANOVA) implemented in the vegan package (v2.7.3). Models included both diet and experimental batch as factors to account for the independent experimental replicates (e.g., DSS and antibiotic experiments), using a formula of the form distance ~ diet + batch.

Correlation analyses were performed using Spearman’s rank correlation coefficient.

Samples were excluded if they were identified as outliers based on a predefined threshold (values exceeding Q3 + 3 × IQR or below Q1 − 3 × IQR within each group).

### 2.10. Data Availability

Data are available from the corresponding author upon request. Sequence data generated and analyzed during the current study are available in the NCBI SRA repository BioProject PRJNA1443396.

## 3. Results

### 3.1. Early-Life Iron Supplementation Induces Long-Lasting Effects After 2 Weeks of Luminal Iron Washout

**Figure 1:**
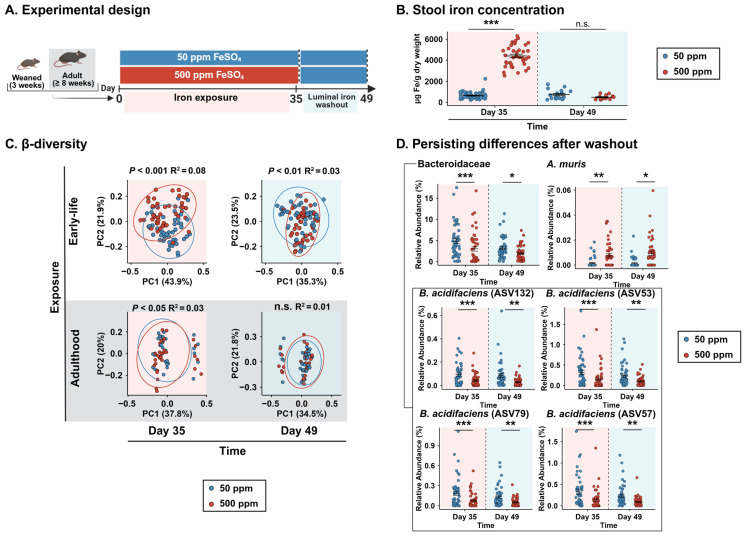
Early-life iron supplementation durably alters gut microbiota composition despite normalization of luminal iron levels. (**A**) Schematic overview of the experimental design up to luminal iron washout. (**B**) Stool iron concentration measured at the end of early-life iron exposure and after the washout period. Bars show mean ± SEM; Mann–Whitney U test. Stool iron measurements after the washout phase were unavailable for a subset of mice due to insufficient fecal material. (**C**) Principal coordinate analysis (PCoA) of weighted UniFrac distances at the end of exposure and after washout in mice exposed during early life and adulthood. Ellipses represent 95% confidence intervals around group centroids; PERMANOVA testing the effect of diet while accounting for batches. (**D**) Relative abundance of features with differences persisting from early-life exposure through washout (DESeq2 Wald test with FDR correction). Bars show mean ± SEM; n = 47 mice/group. * *p* < 0.05, ** *p* < 0.01, *** *p* < 0.001; n.s.: not significant.

To model early-life dietary iron excess, we fed weaned (3-week-old) C57BL/6 mice either an iron-sufficient (50 ppm) or iron-excess (500 ppm) diet for 5 weeks, until adulthood (8 weeks of age). All mice were then put on the iron-sufficient diet for 2 weeks to normalize luminal iron levels (Figure 1A). This washout period allowed us to determine if effects induced by excess iron would persist after luminal iron levels returned to normal.

We first assessed the efficacy of our model by measuring stool iron content at the end of the dietary iron exposure and at the end of the washout period (Figure 1B). At the end of the exposure, mice fed the iron-excess diet displayed markedly higher stool iron levels than those fed the iron-sufficient diet (~7-fold increase, *p* < 0.001). After two weeks on the iron-sufficient diet, stool iron levels in the mice previously exposed to excess iron returned to baseline, with no significant differences between groups (*p* = 0.15).

**Figure A1:**
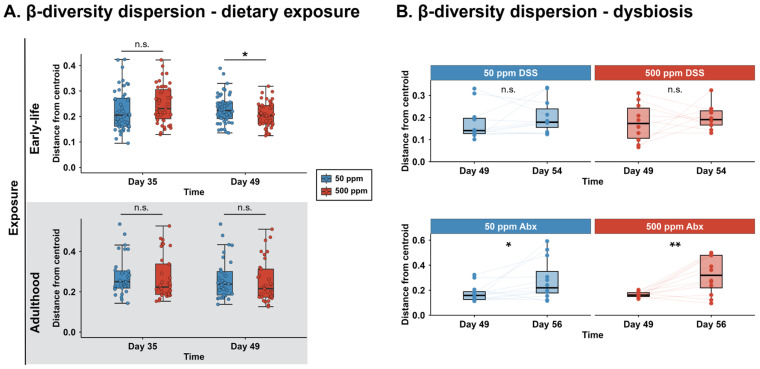
β-diversity dispersion for β-diversity measures prior to dysbiosis induction and measures in during dysbiosis induction. (**A**) β-diversity dispersion (distance from centroid) reported for mice exposed during early-life and adulthood, at end of dietary exposure (Day 35) and at the end of the washout period (Day 49). Bars show mean ± SEM; n = 47 mice/group. (**B**) β-diversity dispersion for mice exposed during early-life between pre-dysbiosis (Day 49) and end of dysbiosis induction (Day 54/56). Lines connect paired samples from the same mouse; DSS: n = 11–10/group, Abx: n = 12/group. ANOVA. * *p* < 0.05, ** *p* < 0.01, n.s.: not significant.

To assess if this iron excess induced differences in gut microbiota community structure, PCoA was performed. A significant separation of bacterial communities between the 50 ppm and 500 ppm groups was observed at the end of the early-life-exposure period (*p* < 0.001, R^2^ = 0.08). This difference remained detectable at the end of the washout period (*p* < 0.01, R^2^ = 0.03). Analysis of multivariate dispersion at this timepoint revealed a significant difference in the average distance to the centroid between groups (*p* < 0.05), with the 500 ppm group exhibiting significantly lower dispersion than the 50 ppm group (Figure A1A).

To assess if the observed effect was specific to mice exposed during early life, we performed the same analysis on mice that were exposed during adulthood and found similar changes at the end of the exposure period (*p* < 0.05, R^2^ = 0.03). However, these changes did not persist after two weeks of luminal iron washout (*p* = 0.32, R^2^ = 0.01), contrasting with the findings seen with early-life supplementation (Figure 1C).

**Figure A2:**
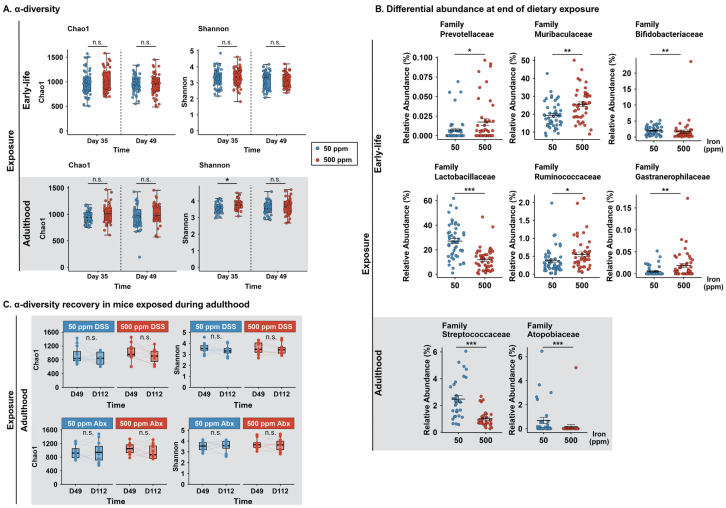
Impact of early-life and adult iron exposure on gut microbiota diversity and composition. (**A**) α-diversity at the end of iron exposure and after the washout period in early-life- and adult-exposed mice; Student’s *t*-test or Mann–Whitney U test if data were not normally distributed; n = 47/group. (**B**) Relative abundance of bacterial families differentially abundant at the end of dietary exposure in early-life- and adult-exposed mice; DESeq2 Wald test with FDR correction; early-life: n = 47/group, adulthood: n = 40–39/group. (**C**) Change in α-diversity between pre-dysbiosis (Day 49) and endpoint (Day 112). Lines connect paired samples from the same mouse; Pairwise *t*-test; DSS: n = 12–11/group, Abx: n = 12/group. (**A**–**C**) Bars show mean ± SEM. * *p* < 0.05, ** *p* < 0.01, *** *p* < 0.001; n.s.: not significant.

No significant differences in α-diversity were observed at either timepoint for the early-life experiment, but in the adult mice, those that received the 500 ppm diet had an increased Shannon index in comparison to those that received the 50 ppm diet, at the end of the dietary exposure. However, that difference did not persist (Figure A2A).

To further characterize these community-level differences, we identified several bacterial families that were differentially abundant between the 50 ppm and 500 ppm groups at the end of the early-life-exposure period (Figure A2B). Among these, only *Bacteroidaceae* remained significantly reduced in mice previously exposed to the 500 ppm iron diet after the washout period. Among this family, four ASVs identified as *Bacteroides acidifaciens* were all lower in the 500 ppm group. An ASV identified as *Adlercreutzia muris* was also persistently different across Day 35 to Day 49 (Figure 1D).

Taken together, these data show that early-life iron supplementation alters gut microbiota composition during the exposure period and that some of these differences persist. Despite comparable stool iron concentrations following the washout period, microbial community structure remained significantly different between groups, with *Bacteroidacea* as the only taxon displaying sustained differences. Such an effect was not observed when iron supplementation was given during adulthood, confirming the effect is specific to early-life exposure.

### 3.2. Early-Life Iron Supplementation Exacerbates DSS-Induced Colitis Symptoms

Following the iron exposure and luminal iron washout periods, we induced dysbiosis through two different methods: through DSS-induced acute colitis, by administration of 0.75% DSS in drinking water for 5 days, or through administration of an antibiotic cocktail consisting of metronidazole (500 mg/L) and ciprofloxacin (200 mg/L) for 7 days.

**Figure 2:**
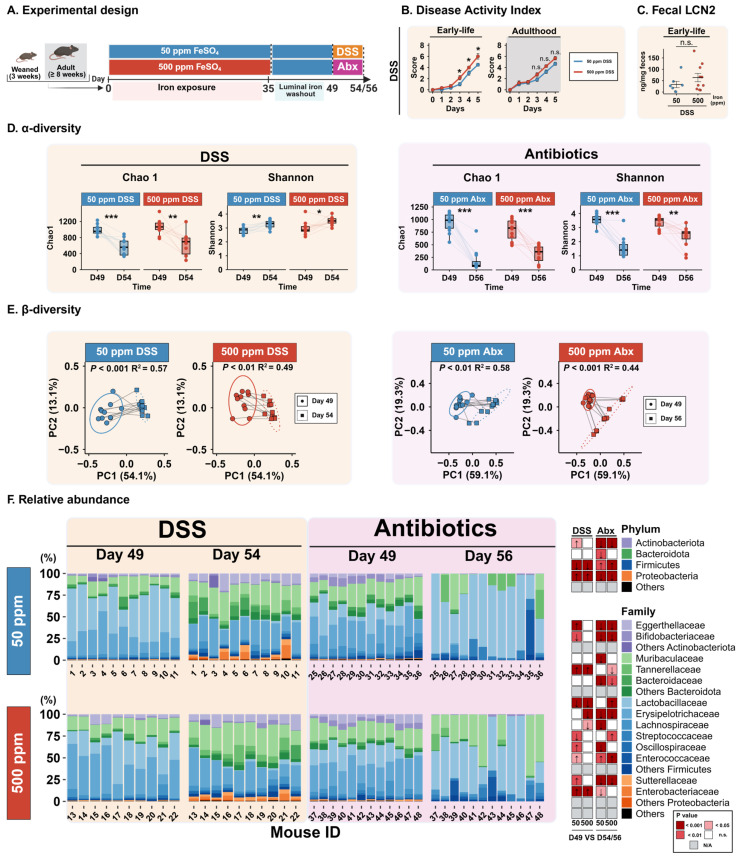
Acute colitis severity and microbiota disruption during DSS- and antibiotic-induced dysbiosis under distinct iron exposures. (**A**) Schematic of the experimental design up to dysbiosis induction. (**B**) Disease activity index (DAI) in DSS-treated mice exposed during early life or adulthood. Bars show mean ± SEM; Mann–Whitney U test. (**C**) Fecal LCN2 in DSS-treated mice exposed during early-life. Bars show mean ± SEM; Student’s *t*-test. Some measurements were unavailable for a subset of mice due to insufficient fecal material. (**D**) Change in Chao1 richness and Shannon diversity between Day 49 (end of washout) and Day 54/56 (end of dysbiosis) for DSS- and antibiotic-treated mice. Lines connect samples from the same mouse; paired *t*-test. (**E**) PCoA of weighted UniFrac distances comparing Day 49 and Day 54/56. Lines connect samples from the same mouse. Ellipses show 95% confidence intervals; PERMANOVA. (**F**) Gut microbiota composition at phylum and family level at Day 49 and Day 54/56. Stacked bars show individual mice; colors indicate bacterial families grouped by phylum; heatmap highlights differentially abundant taxa (arrows indicate direction of change at Day 54/56 relative to Day 49; colors indicate significance). Some samples yielded no sequencing reads and were therefore excluded from the microbiota data panels; DSS: n = 11–10/group, Abx: n = 12/group; DESeq2 Wald test, FDR corrected. * *p* < 0.05, ** *p* < 0.01, *** *p* < 0.001; n.s.: not significant.

To confirm successful induction of acute colitis, disease activity index (DAI) scores were calculated based on body weight loss, stool consistency, and rectal bleeding. DSS exposure resulted in increasing DAI scores during the 5 Days of DSS exposure in both early-life- and adult-life-exposed mice, confirming the effectiveness of the model. In the early-life-exposure experiment, mice exposed to 500 ppm iron displayed significantly higher DAI scores compared to mice exposed to 50 ppm iron (*p* < 0.05; Figure 2B). In contrast, no difference in DAI scores was observed between dietary groups in mice exposed during adulthood. To verify if higher DAI scores reflected increased intestinal inflammation, we measured fecal levels of the pro-inflammatory marker lipocalin-2 (LCN2) on Day 4 of DSS treatment and found no statistically significant differences (Figure 2C).

### 3.3. DSS and Antibiotics Induce Distinct Gut Microbiota Compositional Shifts

To characterize the dysbiosis induced by DSS and antibiotics, gut microbiota composition was compared between the end of the washout period (Day 49) and the end of dysbiosis induction (Day 54 for DSS and Day 56 for antibiotics).

Analysis of α-diversity revealed that DSS exposure significantly reduced microbial richness, as measured by the Chao1 index (50 ppm DSS: *p* < 0.001; 500 ppm DSS: *p* < 0.01), but increased overall diversity assessed by the Shannon index (50 ppm DSS: *p* < 0.01; 500 ppm DSS: *p* < 0.05). Antibiotic treatment also resulted in a pronounced decrease in Chao1 richness (50 ppm Abx: *p* < 0.001; 500 ppm Abx: *p* < 0.001) but showed a decrease in Shannon diversity as well (50 ppm Abx: *p* < 0.001; 500 ppm Abx: *p* < 0.01; Figure 2D). Notably, in the antibiotic-treated groups, mice exposed to 50 ppm iron exhibited greater losses in richness and diversity compared to the 500 ppm group, exhibiting a nearly two-fold-greater loss in Shannon index (∆Shannon: 1.98 ± 0.22 vs. 1.02 ± 0.25, *p* < 0.01); in contrast, DSS-induced changes were of similar magnitude across both diets (Table A2).

β-diversity analysis based on weighted UniFrac distances demonstrated significant shifts in gut microbial community structure following both DSS and antibiotic treatments (all *p* < 0.01), with clear separation between Day 49 and Day 54/56 samples (Figure 2E). Notably, analysis of multivariate dispersion revealed that only in the antibiotic-treated mice, the average distance from the centroid was significantly higher at Day 56 compared to Day 49 in both diet groups (*p* < 0.05).

To identify the taxa underlying these community shifts, differential abundance analyses were performed at the phylum and family levels, comparing Day 49 with Day 54/56 within each treatment group with a longitudinal model (Figure 2F). In DSS-treated mice, dysbiosis was characterized by a significant decrease in Firmicutes and an increase in Proteobacteria (all *p* < 0.001). The reduction in Firmicutes was primarily driven by decreases in *Lactobacillaceae* family (*p* < 0.001) while increases in Proteobacteria were driven by *Enterobacteriaceae* increases (*p* < 0.001). *Tannerellaceae* showed a significant increase (all *p* < 0.001). Despite some diet-specific differences, overall taxonomic shifts in DSS-treated mice were consistent across dietary iron groups.

In contrast, antibiotic-induced dysbiosis resulted in markedly different compositional changes. Proteobacteria and Actinobacteria were strongly depleted in both diet groups (all *p* < 0.001), while Firmicutes was the only phylum that increased following antibiotic exposure (all *p* < 0.01). Decreases in Proteobacteria and Actinobacteria were driven by reductions across their main representative families, including *Eggerthellaceae*, *Bifidobacteriaceae*, and *Sutterellaceae* (all *p* < 0.001). Changes within Firmicutes were more heterogeneous, with decreases in *Erysipelotrichaceae*, *Lachnospiraceae*, and *Oscillospiraceae* (all *p* < 0.001), alongside increases in *Lactobacillaceae*, *Streptococcaceae*, and *Enterococcaceae* (all *p* < 0.01), depending on the dietary group (Figure 2F).

Diet-specific differences were striking within Bacteroidota members. The phylum significantly decreased only in mice exposed to 50 ppm iron (*p* < 0.01), with no significant change observed in the 500 ppm group. This effect was primarily driven by a reduction in *Muribaculaceae*, which was significant only in the 50 ppm Abx group (*p* < 0.001). In contrast, *Bacteroidaceae* decreased in both diet groups (all *p* < 0.01), whereas *Tannerellaceae* decreased exclusively in the 500 ppm Abx group (*p* < 0.05).

Together, these results demonstrate that early-life iron supplementation may exacerbate DSS-induced acute colitis severity, an effect that was not observed following supplementation during adulthood. While DSS- and antibiotic-induced dysbiosis both resulted in marked alterations in gut microbiota diversity and composition, the nature of these shifts differed substantially between models. DSS-induced microbiota changes were largely consistent across both diets, whereas antibiotic-induced dysbiosis was associated with more pronounced losses in richness and diversity in 50 ppm exposed mice, particularly driven by a stronger depletion of Bacteroidota and especially *Muribaculaceae*.

### 3.4. Early-Life Iron Exposure Alters Recovery from Dysbiosis

**Figure 3:**
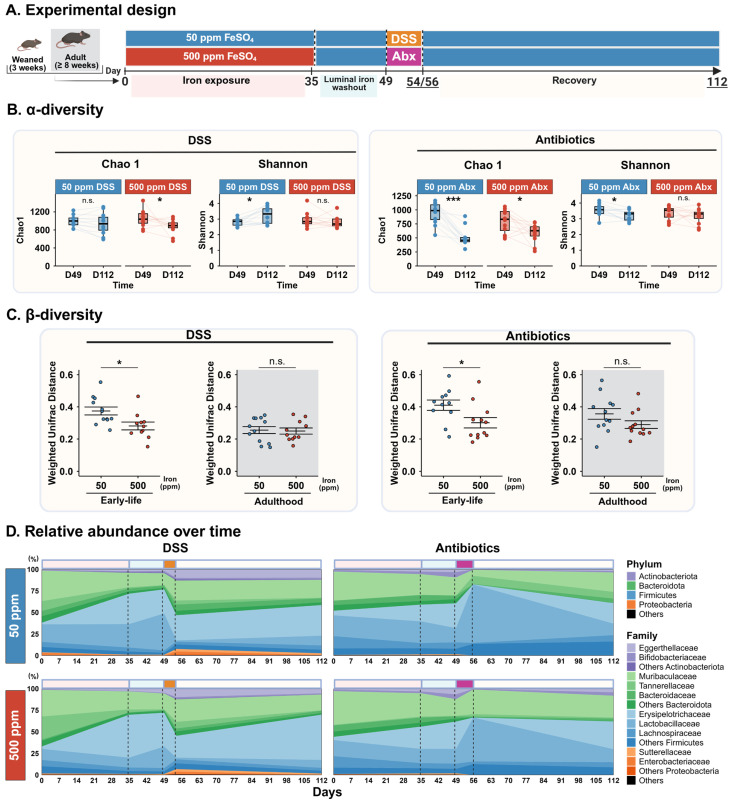
Recovery from dysbiosis is influenced by early-life dietary iron exposure. (**A**) Schematic of the experimental design. (**B**) Chao1 richness and Shannon diversity at Day 49 (end of washout) and Day 112 (end of experiment) for DSS- and antibiotic-treated mice. Bars show mean ± SEM; lines connect samples from the same mouse; paired *t*-tests. (**C**) Weighted UniFrac distances calculated per mouse between Day 49 and Day 112, shown separately for 50 ppm and 500 ppm exposed mice, DSS and antibiotic models, and early- versus adult-life exposure. Bars show mean ± SEM; Student’s *t*-test; DSS-early-life: n = 12–11/group, DSS-adulthood: n = 12–11/group, Abx-early-life: n = 12/group, Abx-adulthood: n = 12/group. (**D**) Average relative abundance of bacterial families over time for each treatment group. Colored horizontal boxes indicate experimental phases; dashed lines mark diet or treatment change. * *p* < 0.05, *** *p* < 0.001; n.s.: not significant.

To evaluate recovery from induced gut dysbiosis, microbial diversity at the end of the washout period (Day 49) was compared to the end of the recovery phase (Day 112). α-diversity was first assessed using the Chao1 richness and Shannon diversity indices (Figure 3B). In DSS-treated mice, a significant change in richness was observed only in the 500 ppm group (Chao1, *p* < 0.05), whereas a significant increase in diversity was detected only in the 50 ppm group (Shannon, *p* < 0.05) relative to baseline. In antibiotic-treated mice, both groups exhibited persistently reduced richness compared to baseline, with a more pronounced decrease in the 50 ppm group (50 ppm: *p* < 0.001; 500 ppm: *p* < 0.05). For the Shannon index, a significant difference was again observed only in the 50 ppm group (*p* < 0.05). On the other hand, mice exposed during adulthood did not show any changes in α-diversity (Figure A2C).

To determine whether recovery trajectories differed as a function of early-life iron exposure, we extracted intra-individual weighted UniFrac distances for each mouse between their baseline (Day 49) and the end of recovery (Day 112) (Figure 3C). Specifically, within the DSS-treated mice, mice exposed to 50 ppm iron remained 33% more distant from their baseline composition than those in the 500 ppm group (0.37 ± 0.025 vs. 0.28 ± 0.024, *p* < 0.05). A similar trend was observed following antibiotic-induced dysbiosis, where the 50 ppm group exhibited a 41% greater distance from baseline compared to 500 ppm mice (0.43 ± 0.033 vs. 0.30 ± 0.032, *p* < 0.05) (Table A3).

To confirm if this effect was early-life-specific, we performed the same analysis for mice exposed during adulthood. No significant differences were observed between 50 ppm and 500 ppm adult mice following DSS challenge. While adult mice exposed to antibiotics showed a slight trend toward higher dissimilarity in the 50 ppm group (0.35 + 0.033 vs. 0.29 + 0.024; a 23% increase), this did not reach statistical significance (*p* = 0.11).

To gain a general understanding of recovery dynamics, we plotted the mean relative abundance of major phyla and families across the experimental timeline in early-life-exposed mice that received DSS or antibiotics (Figure 3D). In DSS-exposed mice, Proteobacteria levels remained significantly elevated relative to baseline, despite a notable decline following their initial bloom after DSS exposure. Furthermore, mice exposed to 500 ppm returned closer to their baseline Firmicutes abundance than those in the 50 ppm group. Differences were particularly visible with the mice exposed to antibiotics, as *Muribaculaceae* mean relative abundance remained relatively stable during the time course of the experiment in the 500 ppm exposed mice compared to the 50 ppm group.

Taken together, these results indicate that recovery from gut dysbiosis is incomplete eight weeks after the end of dysbiosis induction and is modulated by early-life iron exposure. While both dietary groups exhibited persistent alterations in community structure, mice exposed to the iron-sufficient diet during early life showed a greater divergence from their pre-dysbiosis microbiota compared to iron-excess-exposed mice, while adult-exposed mice did not show any significant differences overall, suggesting distinct recovery trajectories depending on early-life iron supplementation.

### 3.5. Enrichment of Ligilactobacillus murinus Correlates with Elevated Fecal Succinate Levels

**Figure 4:**
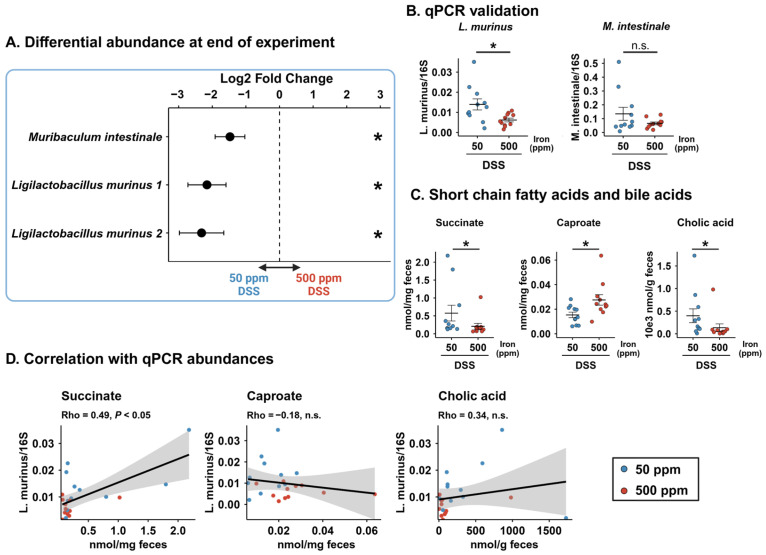
Differential abundance of *Ligilactobacillus murinus* at the experimental endpoint correlates with shifted succinate levels following recovery from DSS-induced dysbiosis. (**A**) Log2 fold change in significant differentially abundant ASVs annotated at the species level, comparing 50 ppm and 500 ppm DSS-exposed mice. Points show LFC, bars show LFC standard error; FDR-corrected Wald test. (**B**) qPCR validation of selected bacterial species; Student’s *t*-test. (**C**) Stool concentrations of SCFAs and bile acids that differed significantly between groups; Mann–Whitney U test. (**D**) Spearman correlation between *L. murinus* qPCR abundance and stool caproate, succinate, and cholic acid. Shaded areas represent 95% confidence intervals; Spearman’s rank correlation (ρ). Bars show mean ± SEM; n = 11/group. One mouse in the 50 ppm group with cholic acid and succinate measures > Q3 + 3 × IQR was excluded. * *p* < 0.05; n.s.: not significant.

**Figure A3:**
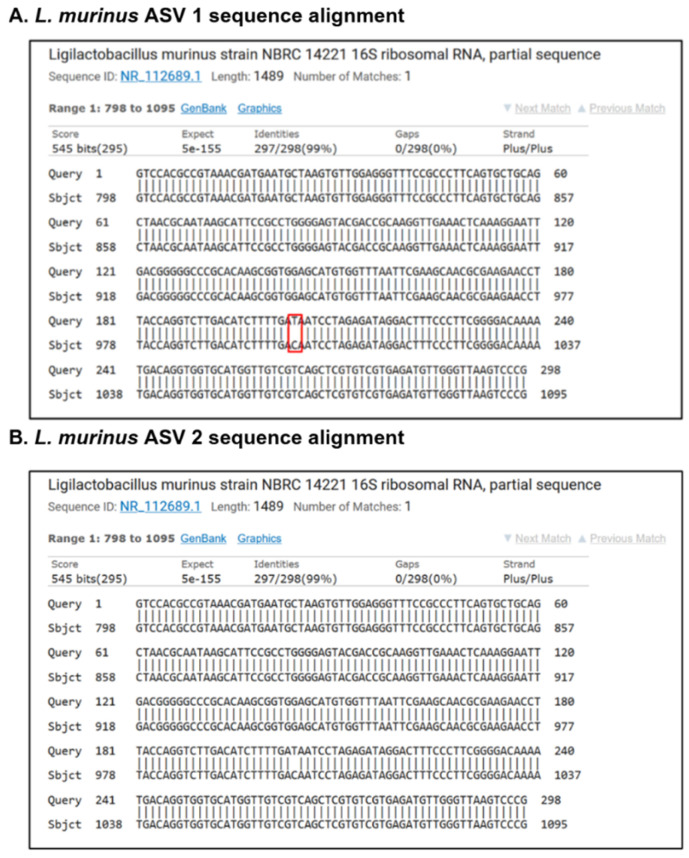
Two differentially abundant ASVs share high sequence similarity with the V5–V6 region of 16S rRNA genes of *Ligilactobacillus murinus*. (**A**,**B**) Alignments of the two ASVs with the *L. murinus* V5–V6 region. The red box indicates a mismatch in the alignment. Alignments were performed using BLASTn [38] against the NCBI rRNA/ITS database [39].

**Figure A4:**
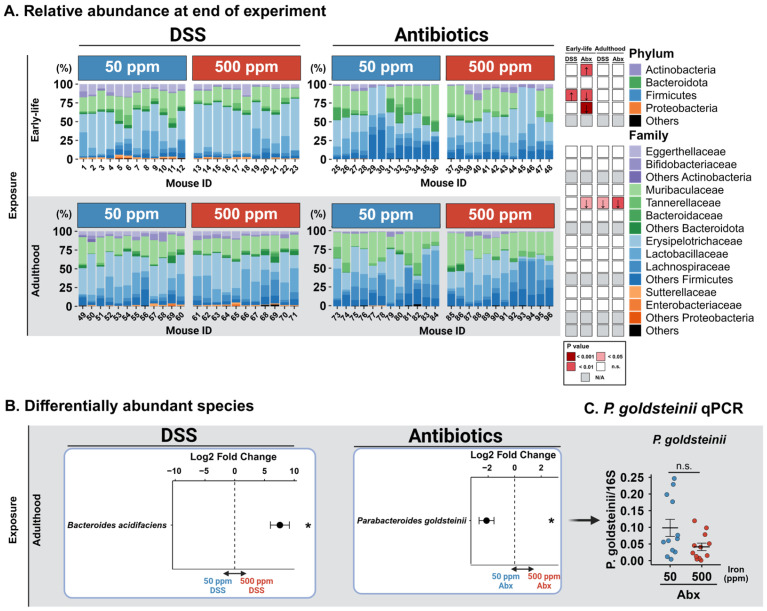
Gut microbiota composition at last timepoint for mice exposed during early life and adulthood. (**A**) Gut microbiota composition at phylum and family level at endpoint in mice exposed to iron during early life or adulthood. Stacked bars show individual mice; colors indicate bacterial families grouped by phylum (legend). Heatmap highlights differentially abundant taxa; arrows indicate direction of change (↑ increased, ↓ decreased in 500 ppm vs. 50 ppm); colors indicate significance; DESeq2 Wald test, FDR corrected; DSS-early-life: n = 12–11/group, DSS-adulthood: n = 12–11/group, Abx-early-life: n = 12/group, Abx-adulthood: n = 12/group. (**B**) Log2 fold change in differentially abundant ASVs annotated at the species level in adult-exposed mice comparing 50 ppm and 500 ppm, in either DSS or antibiotic model, at endpoint. Points show LFC; bars show LFC standard error; DESeq2 Wald test, FDR corrected. (**C**) qPCR abundance of *Parabacteroides goldsteinii* in adult-exposed mice treated with antibiotics, at endpoint. Bars show mean ± SEM; Mann–Whitney U test; n = 12/group. * *p* < 0.05; n.s.: not significant.

To assess what differences these distinct recovery trajectories would induce on the gut microbiota, we compared the 50 ppm and 500 ppm treatment groups at the end timepoint. In the DSS experiment, differential abundance analysis revealed a significant increase in specific taxa in the 50 ppm group relative to the 500 ppm group (Figure 4A). Notably, one ASV annotated as *Muribaculum intestinale* and two distinct ASVs that we annotated as *Ligilactobacillus murinus* using BLAST (Figure A3) exhibited higher abundance in 50 ppm DSS-treated mice relative to 500 ppm DSS-treated mice (all *p* < 0.05). These findings were further validated via qPCR, which confirmed a significant increase in *L. murinus* abundance in the 50 ppm group (*p* < 0.01), while *M. intestinale* did not reach statistical significance (Figure 4B). In mice exposed during adulthood, we did not observe such taxonomical shifts (Figure A4B).

These taxonomic shifts were accompanied by distinct alterations in the fecal metabolome. Mice in the 500 ppm DSS group exhibited significantly higher concentrations of stool caproate (*p* < 0.05), while the 50 ppm DSS group showed increased concentrations of succinate (*p* < 0.05) and cholic acid (*p* < 0.01) (Figure 4C). To explore the potential functional relationship between the depleted microbiota and these metabolites, we performed Spearman correlation analyses (Figure 4D). We observed a positive correlation between the qPCR abundance of *L. murinus* and succinate (Rho = 0.49, *p* < 0.05), suggesting a link between the two.

These results show that in the DSS model, the different recovery trajectories result in a taxonomically and functionally distinct gut microbiota. Mice exposed to the 50 ppm diet were marked by a bloom of *L. murinus* and associated with a shifted functional profile, notably for caproate, succinate and cholic acid, with *L. murinus* abundance positively correlating with stool succinate.

### 3.6. Divergent Taxonomic and Inferred Metabolic Profiles During Post-Antibiotic Recovery Following Early-Life Iron Exposure

**Figure 5:**
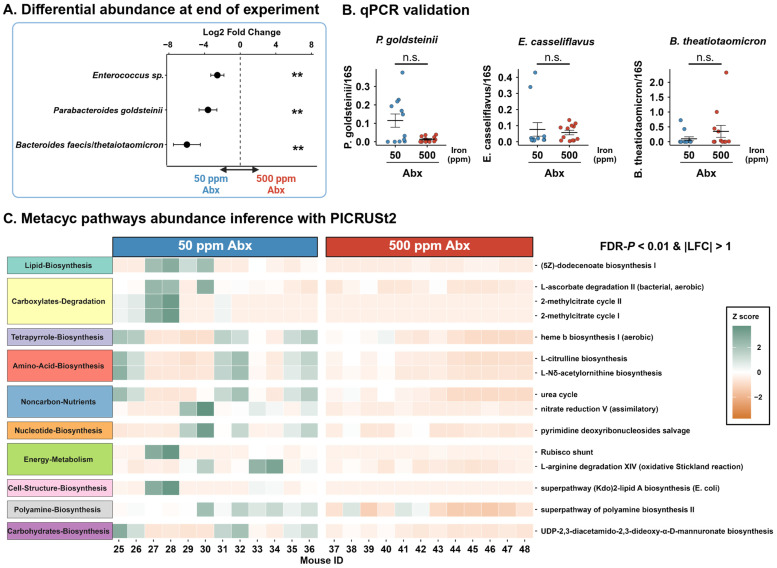
PICRUSt2 infers important functional differences at endpoint in mice that received antibiotics. (**A**) Log2 fold change in significant differentially abundant ASVs annotated at the species level, comparing 50 ppm and 500 ppm antibiotic-exposed mice. Points show LFC, bars show LFC standard error; FDR-corrected Wald test. (**B**) qPCR validation of selected bacterial species; Mann–Whitney U test. (**C**) Heatmap of Z-scores for significantly different MetaCyc pathways inferred using PICRUSt2; FDR-corrected Wald test; n = 12/group. ** *p* < 0.01; n.s.: not significant.

Mice that received antibiotics exhibited higher inter-individual variability in microbial community composition within groups than those treated with DSS. In each group, two mice retained a highly imbalanced gut microbiota with a depleted proportion of Bacteroidota (Mice 29, 30, 45, 46; Figure A4A). Taxonomic differences were marked by a higher relative abundance of Actinobacteriota and lower amounts of Firmicutes in 500 ppm exposed mice. Proteobacteria were only detected in two mice from the 50 ppm group. We were able to identify three ASVs annotated at the species level, but two of them had ambiguous taxonomic assignments (Figure 5A). One was assigned to multiple *Enterococcus* species, for which the 16S region is highly conserved. Another ASV was assigned to two highly similar *Bacteroides* species (*Bacteroides faecis* and *Bacteroides thetaiotaomicron*). *Parabacteroides goldsteiini* was also found to be differentially abundant in mice exposed during adulthood (Figure A4B). Taking that into account, we chose our primers focusing on specific species which were more likely to be found in wild-type C57BL/6 mice and for which there were documented primers in the literature, respectively, *Enterococcus casseliflavus* and *Bacteroides thetaiotaomicron*. We cannot confirm these species to be differentially abundant as none of our qPCRs reached significance (Figure 5B). *P. goldsteinii* was also found to be differentially abundant in mice exposed during adulthood but did not reach significance when validating with qPCR either (Figure A4B,C).

**Figure A5:**
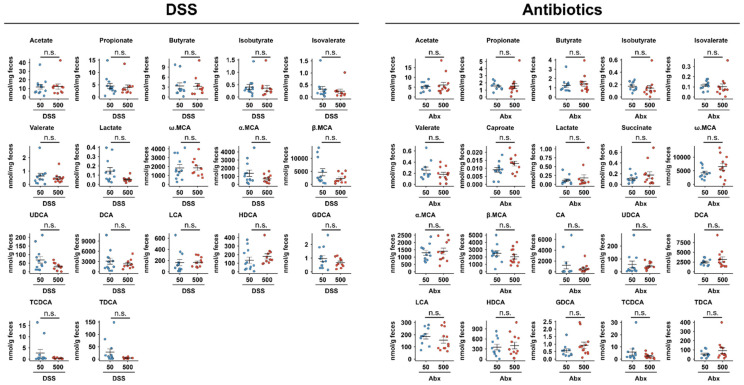
Stool SCFAs and BAs at end of experiment. Stool SCFAs and bile acids were measured at the end of the experiment in mice exposed during early life to 50 ppm or 500 ppm iron, treated with either DSS or antibiotics. Statistical analysis: Student’s *t*-test, Welch’s *t*-test, or Mann–Whitney U test, depending on data distribution. n.s.: not significant. DSS: n = 12–11/group, Abx n = 11–12/group. Some measurements were unavailable for one mouse in the 50 ppm Abx group due to insufficient fecal material.

We still sought to determine if the taxonomic shifts identified using differential abundance analysis translated into functional disparities between the two groups. Our targeted metabolomics approach did not yield any significant results (Figure A5). Using PICRUSt2 to infer metabolic pathway abundances from taxonomic profiles, we mapped the sequences to the MetaCyc database (Figure 5C). As many different metabolic pathways were inferred as differentially abundant between our two groups, we selected the ones that had an absolute LFC > 1 and a FDR *p* < 0.01. All pathways were detected as being more abundant in the 50 ppm group. Most differentially abundant pathways were related to carboxylate degradation, energy metabolism and amino acid biosynthesis, while overall effects were variable within the same group and were often driven by a subset of mice.

Collectively, these data suggest that recovery from antibiotic exposure after iron supplementation in early life results in distinct taxonomic shifts and altered inferred metabolic pathways, particularly in the 50 ppm group. However, high within-group variability confounded the identification of distinct species distributions, and targeted metabolomics revealed no significant differences in metabolite concentrations between groups.

## 4. Discussion

In this study, we provide evidence that early-life iron supplementation shapes the development of the gut microbiota and influences its subsequent ability to recover from dysbiosis in adulthood. Our findings demonstrate that, following five weeks of an iron-excess diet and a subsequent two-week washout period, early-life exposure induces lasting alterations that persist even after luminal iron concentrations have normalized (β-diversity, PERMANOVA *p* < 0.01). Furthermore, mice exposed to excess iron during early life exhibited significantly greater microbiota stability during antibiotic challenge, showing nearly two-fold less loss in Shannon diversity compared with the sufficient iron group. Across two distinct models of dysbiosis, we further demonstrate that early-life iron exposure altered the recovery trajectory; specifically, excess iron mice returned significantly closer to their baseline microbial composition, exhibiting 33% (DSS) and 41% (antibiotics) lower weighted UniFrac distances compared with sufficient iron mice. Importantly, these observations could not be recapitulated when iron supplementation occurred during adulthood, further supporting that the observed effects on the gut microbiota are specific to early-life exposure.

Early-life iron exposure strongly reshaped the developing microbiota, decreasing the abundance of *Lactobacillaceae* and *Bacteroidaceae*, two bacterial families with recognized immunomodulatory potential [49,50,51]. Reduction in these key commensals may represent a significant shift in the functional landscape of the gut, as they are essential for priming mucosal immunity and maintaining microbial–host homeostasis through the production of immunomodulatory metabolites such as SCFAs or indole derivatives [52,53]. Members of the *Lactobacillacaeae* family have been reported to thrive under low-iron conditions due to their low iron requirements and to decrease under high-iron conditions in both animal and humans [54], though less is known regarding the mechanisms lowering their abundance under excess iron conditions. To our knowledge though, no study has reported members of the family *Bacteroidaceae* to be reduced under high-iron conditions. Here we reported several ASVs annotated as *Bacteroides acidifaciens* that remained lower in mice exposed to excess iron even after returning to an iron-sufficient diet. *B. acidifaciens* has previously been reported to alleviate DSS-induced colitis symptoms in a fecal microbiota transplantation mouse model [55].

Although mice exposed to excess iron during early life displayed more severe DSS-induced acute colitis symptoms, we did not observe a significant correlation between disease activity index (DAI) and the abundance of *B. acidifaciens*. While luminal iron availability has also been shown to exacerbate DSS-induced colitis in mice [56,57,58,59], fecal iron concentrations in our study returned to baseline after two weeks under the iron-sufficient diet, suggesting that the observed phenotype was unlikely driven by residual luminal iron. Instead, the interaction between early-life microbiota composition and host immune development may contribute to this susceptibility, as increasing evidence links early-life microbial perturbations to ulcerative colitis risk later in life [60,61,62], whereby commensal-derived signals during a critical developmental window are required to program the differentiation of regulatory T cells (Tregs) and establish mucosal tolerance [63]. This susceptibility may be further compounded by the direct toxicity of iron; a recent study found that a 5000 ppm iron diet administered to weaned C57BL/6 mice for 7 weeks induced a colitis-like state by activating colonic ferroptosis and depleting beneficial taxa such as *Lactobacillus* [64].

A key finding of this study was the different recovery trajectories observed following dysbiosis. Early-life iron exposure appears to influence the resilience of the microbial community, defined as its capacity to withstand disturbance and return to its baseline configuration [65]. Mice exposed to excess iron during early life recovered to a state mirroring the stability seen in mice exposed during adulthood, while those that were maintained on the iron-sufficient diet during early life showed a greater compositional drift. Similar recovery dynamics were observed across both dysbiosis models used in this study (DSS-induced inflammation and antibiotic-mediated microbiota depletion) indicating that early-life iron exposure broadly affects microbiota resilience rather than responses to a single perturbation. However, the two models exhibited distinct primary responses. Specifically, when given antibiotics, mice exposed to excess iron during early life displayed significantly higher resistance to perturbation, evidenced by an approximately two-fold reduction in α-diversity loss and a mitigated depletion of the *Muribaculaceae* family compared to their iron-sufficient counterparts. Conversely, both diet groups showed similar microbial shifts when given DSS, suggesting that while the early-life iron effect on recovery (resilience) was the same across stressors, its impact on initial resistance may be perturbation-specific.

These findings may be explained by ecological priority effects, whereby early environmental conditions and colonization order influence long-term community assembly [66]. Excess iron during the neonatal “window of opportunity” may act as a strong environmental filter, selecting for bacterial taxa adapted to high-iron conditions, and may constrain subsequent community assembly, stabilizing the resulting microbial structure. Long-term effects have also been observed in rats, in which postnatal iron supplementation induced microbial alterations that persisted into adulthood. Although the taxonomic responses to ferrous sulfate in these models differed from those observed in our study, these findings nonetheless suggest that early-life iron-induced alterations to the gut microbiota can persist into adulthood. This phenomenon is consistent with the concept of priority effects, whereby early environmental filters shape subsequent successional trajectories [67]. Consistent with this, the *Muribaculaceae* family, enriched in mice exposed to excess iron during early-life, remained relatively stable in this group, even during antibiotic treatment. In contrast, its abundance markedly decreased in mice that received the iron-sufficient diet when antibiotics were given, suggesting that early-life iron exposure may stabilize specific microbial lineages following disturbances.

These results also raise an important question regarding the ecological implications of the observed recovery patterns. The apparent stability of the microbiota in the high-iron group could represent either a robust return to homeostasis or a constrained ecological trajectory resulting from early environmental filtering. In contrast, the compositional drift noted in the mice exposed to sufficient iron during early life may reflect greater ecological flexibility, where perturbation allows the community to transition toward an alternative stable configuration.

To further explore potential functional outcomes, we examined microbiota composition and function at the endpoint of each dysbiosis model. In DSS-treated early-life-exposed mice, we observed a bloom of *L. murinus*, a widely studied candidate probiotic in both mouse and human hosts, with multiple reported functions such as butyrate production [68], immunomodulatory activity [69], epithelial cell renewal in the gut [70] and intestinal repair [71]. The expansion of *L. murinus* may be interpreted in several ways. It could reflect the occupation of an advantageous niche, potentially driven by the priority effects described above, as the abundance of *Lactobacillaceae* was markedly reduced following five weeks of excess iron exposure. Alternatively, this proliferation may represent an immune-driven compensatory response aimed at promoting intestinal repair [72,73], given that DSS induces epithelial damage in the colon. Interestingly, elevated succinate levels, which have been associated with imbalances between succinate producers and consumers in DSS and IBD models [74], also correlated with *L. murinus*. Although *L. murinus* is not known to directly produce succinate, this co-occurrence is consistent with a previous report describing a simultaneous increase in both the species and the metabolite during the recovery phase following DSS exposure [75]. The absence of this signature in the excess iron group is particularly striking given their more pronounced acute symptoms during DSS exposure, highlighting potential differences in recovery dynamics.

Functionally, targeted metabolomics revealed largely similar profiles between groups. However, taxonomic-based functional inference using PICRUSt2 suggested underlying differences, with several pathways predicted to be more abundant in mice exposed to sufficient iron during early life. Notably, enrichment of the UDP-2,3-diacetamido-2,3-dideoxy-α-D-mannuronate biosynthesis pathway, which is involved in the production of lipopolysaccharides (LPS) components [76], may indicate a shift toward Gram-negative-associated functions and a potentially increased inflammatory capacity. In parallel, there was enrichment of the 2-methylcitrate cycle, a well-characterized pathway for microbial propionate catabolism and detoxification [77]. Polyamine biosynthesis pathways were also enriched, which are involved in cellular proliferation and mucosal repair [78]. Additionally, enrichment of the pyrimidine deoxyribonucleotide salvage pathway may reflect enhanced nucleotide recycling capacity, a feature associated with bacterial growth and antibiotic stress responses [79]. However, given the absence of corresponding differences in measured metabolites, these functional predictions should be interpreted with caution. PICRUSt2 identifies genomic potential rather than active metabolic flux; therefore, the observed differences may reflect the expansion of specific low-abundance taxa, such as Proteobacteria, without signifying a community-wide metabolic alteration.

It is also worth noting that in our study, iron was administered exclusively as ferrous sulfate, one of the most commonly used formulations. Different iron forms have different bioavailability and reactivity and may differentially affect gut microbial communities [20,29,67]. Accordingly, the effects observed here should be interpreted as specific to ferrous sulfate rather than to iron supplementation broadly. Similarly, in the antibiotic model, dysbiosis was induced using a cocktail targeting both anaerobic and aerobic bacteria, while other antibiotics may induce different perturbations and resulting recovery patterns.

Overall, these findings may support a counterintuitive idea: excess dietary iron during infancy may provide a protective advantage. While iron supplementation is traditionally associated with the expansion of pathogens [80], we did not observe such shift, which may be dependent on the intestinal environment and the gut microbiota composition. In this controlled setting, excess iron stress may have primed the microbiota to become more resilient and better adapted to handle subsequent dysbiotic challenges later in life. Conversely, the lack of mechanistic insights makes it difficult to determine whether early-life iron exposure was ultimately beneficial or detrimental for recovery after dysbiosis, and the increased severity of DSS-induced colitis symptoms together with the enrichment of *L. murinus* only in mice exposed to sufficient iron may support a detrimental effect of excess iron exposure. Nevertheless, early-life iron exposure clearly influenced the trajectory of gut microbiota recovery, highlighting the need to further investigate how nutritional iron exposure during critical developmental windows shapes responses to microbiota disturbances later in life. In the future, understanding the underlying mechanisms may help guide more personalized nutritional or microbiota-targeted strategies aimed at minimizing adverse long-term effects on intestinal homeostasis, as currently being explored in large-scale preventive trials [81].

## 5. Conclusions

Our findings highlight that iron availability during early-life microbiota establishment acts as an ecological filter that shapes long-term microbial community structure and resilience to perturbation in adulthood. Given the widespread use of milk formulas and oral iron supplementation during infancy, these results suggest that early-life iron exposure may have lasting implications for host–microbiota interactions and intestinal homeostasis later in life. While this study provides novel insights into the long-term consequences of early-life iron excess, future studies are needed to elucidate the underlying mechanisms and determine whether these microbiota alterations ultimately exert beneficial or detrimental effects on host health.

## Data Availability

The original contributions presented in this study are included in the article. Further inquiries can be directed to the corresponding author.

## Abbreviations

The following abbreviations are used in this manuscript:

Abx	Antibiotics
ASV	Amplicon Sequence Variant
BA	Bile Acid
CD	Crohn’s Disease
DAI	Disease Activity Index
DSS	Dextran Sulfate Sodium
IBD	Inflammatory Bowel Disease
LCN-2	Lipocalin-2
LFC	Log2 Fold Change
LPS	Lipopolysaccharides
PERMANOVA	Permutational Multivariate Analysis of Variance
PCoA	Principal Coordinate Analysis
Ppm	Parts Per Million
RNA	Ribonucleic Acid
rRNA	Ribosomal RNA
SCFA	Short-Chain Fatty Acid
UC	Ulcerative Colitis

## Appendix A

### Appendix A.1

**Table A1:** Primers used to detect bacterial species.

Target Species	Primer Name	Sequence (5′ → 3′)	Source
*Muribaculum intestinale*	M. intestinale F	AAGGCATCTTCTTTCGGCCA	This study (Primer-BLAST)
	M. intestinale R	TGATATTCCGCCTACGCACC	
*Ligilactobacillus murinus*	L. murinus F	AAGAGTTGAGCTGAGCGAACG	[82]
	L. murinus R	CGTAGAAGTTTGGGCCGTGTTT	
*Parabacteroides goldsteiini*	P. goldsteiini F	AGCGTTAAGTAATCCACCTGG	This study (Primer-BLAST)
	P. goldsteiini R	TCCAGAGCTGTCAATATGCG	
*Enterococcus casseliflavus*	E. casseliflavus F	GGAGCTTGCTCCACCGAA	[83]
	E. casseliflavus R	TTTCTTCCATGCGGAAAATAGT	
*Bacteroides thetaiotaomicron*	B. thetaiotaomicron F	GGCAGCATTTCAGTTTGCTTG	[84]
	B. thetaiotaomicron R	GGTACATACAAAATTCCACACGT	

### Appendix A.4

**Table A2:** Magnitude of α-diversity decline in DSS and antibioitc-treated mice between Day 49 and 54/56.

Group	∆ Chao1 (Mean ± SEM)	∆ Shannon (Mean ± SEM)
50 ppm DSS	417.9915 ± 77.67	−0.45 ± 0.11
500 ppm DSS	430.6709 ± 121.64	−0.51 ± 0.18
*p*-value (50 ppm DSS vs. 500 ppm DSS)	0.93	0.77
50 ppm Abx	767.47 ± 67.83	1.98 ± 0.22
500 ppm Abx	482.84 ± 88.21	1.02 ± 0.25
*p*-value (50 ppm Abx vs. 500 ppm Abx)	0.018	0.0056

Quantitative analysis of the reduction in microbial α-diversity (Chao1 and Shannon indices) before giving DSS/Antibiotics (Day 49) to the peak of dysbiosis (Day 54/56). Data are presented as the mean difference (∆) per individual mouse ± SEM. *p*-values denote significant differences in the magnitude of loss between 50 ppm and 500 ppm groups; Student’s *t*-test, Mann–Whitney U test for Shannon index in mice exposed to antibiotics.

### Appendix A.5

**Table A3:** Quantitative comparison of microbial recovery trajectories.

Exposure Period	Dysbiosis Model	Diet Group	Weighted UniFrac Distance (Mean + SEM)	% Difference (50 vs. 500)	*p*-Value
Early life	DSS	50 ppm	0.37 + 0.025	33%	0.014
		500 ppm	0.28 + 0.024	—	
	Antibiotics	50 ppm	0.43 + 0.033	41%	0.013
		500 ppm	0.30 + 0.032	—	
Adult life	DSS	50 ppm	0.25 + 0.022	2.3%	0.84
		500 ppm	0.25 + 0.019	—	
	Antibiotics	50 ppm	0.35 + 0.033	23%	0.11
		500 ppm	0.29 + 0.024	—	

Intra-individual weighted UniFrac distances were calculated for each mouse between baseline (pre-dysbiosis) and the end of the recovery period (Day 112). Values represent the mean distance + SEM, where higher values indicate a greater deviation from the original microbial state. The “% Difference” represents the relative increase in dissimilarity observed in the 50 ppm group compared to the 500 ppm group; Student’s *t*-test.

## References

[1] Jandhyala S.M., Talukdar R., Subramanyam C., Vuyyuru H., Sasikala M., Nageshwar Reddy D. (2015). Role of the normal gut microbiota. World J. Gastroenterol..

[2] Zheng D., Liwinski T., Elinav E. (2020). Interaction between microbiota and immunity in health and disease. Cell Res..

[3] Kim S., Seo S.U., Kweon M.N. (2024). Gut microbiota-derived metabolites tune host homeostasis fate. Semin. Immunopathol..

[4] Cheng X., Tan Y., Li H., Huang J., Zhao D., Zhang Z., Yi M., Zhu L., Hui S., Yang J. (2022). Fecal 16S rRNA sequencing and multi-compartment metabolomics revealed gut microbiota and metabolites interactions in APP/PS1 mice. Comput. Biol. Med..

[5] Loh J.S., Mak W.Q., Tan L.K.S., Ng C.X., Chan H.H., Yeow S.H., Foo J.B., Ong Y.S., How C.W., Khaw K.Y. (2024). Microbiota–gut–brain axis and its therapeutic applications in neurodegenerative diseases. Signal Transduct. Target. Ther..

[6] He Y., Hu H., Liang X., Liang J., Li F., Zhou X. (2025). Gut microbes-muscle axis in muscle function and meat quality. Sci. China Life Sci..

[7] Tiffany C.R., Bäumler A.J. (2019). Dysbiosis: From fiction to function. Am. J. Physiol. Gastrointest. Liver Physiol..

[8] Han Y., Wang Z., Xie J., Yang G., Su M., Wang S., Yang M., Yu H., Li M., Wang L. (2026). Host–gut microbiota interactions in health and disease: Mechanisms and intervention strategies. Front. Microbiol..

[9] Li Y.-K., Li W.-R., Ren H., Xiao C.-L., Guo Z., Luo J.-Q. (2025). Gut microbiome-targeted therapeutics for chronic kidney disease: Comparative efficacy of probiotic and microbial preparations. Inflammopharmacology.

[10] Yatsunenko T., Rey F.E., Manary M.J., Trehan I., Dominguez-Bello M.G., Contreras M., Magris M., Hidalgo G., Baldassano R.N., Anokhin A.P. (2012). Human gut microbiome viewed across age and geography. Nature.

[11] Renz H., Adkins B.D., Bartfeld S., Blumberg R.S., Farber D.L., Garssen J., Ghazal P., Hackam D.J., Marsland B.J., McCoy K.D. (2018). The neonatal window of opportunity-early priming for life. J. Allergy Clin. Immunol..

[12] Sarkar A., Yoo J.Y., Valeria Ozorio Dutra S., Morgan K.H., Groer M. (2021). The Association between Early-Life Gut Microbiota and Long-Term Health and Diseases. J. Clin. Med..

[13] Pantopoulos K., Porwal S.K., Tartakoff A., Devireddy L. (2012). Mechanisms of mammalian iron homeostasis. Biochemistry.

[14] Wu Z., Shao J., Zheng J., Liu B., Li Z., Shen N. (2022). A zero-sum game or an interactive frame? Iron competition between bacteria and humans in infection war. Chin. Med. J. (Engl.).

[15] Katsarou A., Pantopoulos K. (2020). Basics and principles of cellular and systemic iron homeostasis. Mol. Asp. Med..

[16] Seyoum Y., Baye K., Humblot C. (2021). Iron homeostasis in host and gut bacteria-a complex interrelationship. Gut Microbes.

[17] Papanikolaou G., Pantopoulos K. (2005). Iron metabolism and toxicity. Toxicol. Appl. Pharmacol..

[18] Cuisiniere T., Calvé A., Fragoso G., Oliero M., Hajjar R., Gonzalez E., Santos M.M. (2021). Oral iron supplementation after antibiotic exposure induces a deleterious recovery of the gut microbiota. BMC Microbiol..

[19] Iddrisu I., Monteagudo-Mera A., Poveda C., Shahzad M., Walton G.E., Andrews S.C. (2025). A review of the effect of iron supplementation on the gut microbiota of children in developing countries and the impact of prebiotics. Nutr. Res. Rev..

[20] Constante M., Fragoso G., Lupien-Meilleur J., Calvé A., Santos M.M. (2017). Iron Supplements Modulate Colon Microbiota Composition and Potentiate the Protective Effects of Probiotics in Dextran Sodium Sulfate-induced Colitis. Inflamm. Bowel Dis..

[21] Atkins L.A., Spence A.C., Szymlek-Gay E.A. (2023). Iron Nutrition of Pre-Schoolers in High-Income Countries: A Review. Nutrients.

[22] World Health Organization, United Nations Children’s Fund (UNICEF) (2025). Global Breastfeeding Scorecard, 2025: Breastfeeding Rates are Increasing but Improved Support is Needed.

[23] Ljung K., Palm B., Grandér M., Vahter M. (2011). High concentrations of essential and toxic elements in infant formula and infant foods-A matter of concern. Food Chem..

[24] Statistics Canada (2017). Types of Nutrients from Nutritional Supplements-Consumed in the Past Month, by Age Group and Sex, Household Population Aged 1 and Over, Canadian Community Health Survey (CCHS)-Nutrition, Canada.

[25] Jaeggi T., Kortman G.A.M., Moretti D., Chassard C., Holding P., Dostal A., Boekhorst J., Timmerman H.M., Swinkels D.W., Tjalsma H. (2015). Iron fortification adversely affects the gut microbiome, increases pathogen abundance and induces intestinal inflammation in Kenyan infants. Gut.

[26] Zimmermann M.B., Chassard C., Rohner F., N’Goran E.K., Nindjin C., Dostal A., Utzinger J., Ghattas H., Lacroix C., Hurrell R.F. (2010). The effects of iron fortification on the gut microbiota in African children: A randomized controlled trial in Côte d’Ivoire1234. Am. J. Clin. Nutr..

[27] Paganini D., Zimmermann M.B. (2017). The effects of iron fortification and supplementation on the gut microbiome and diarrhea in infants and children: A review. Am. J. Clin. Nutr..

[28] Cuisiniere T., Hajjar R., Oliero M., Calvé A., Fragoso G., Rendos H.V., Gerkins C., Taleb N., Gagnon-Konamna M., Dagbert F. (2025). Initial gut microbiota composition is a determining factor in the promotion of colorectal cancer by oral iron supplementation: Evidence from a murine model. Microbiome.

[29] Pantopoulos K. (2024). Oral iron supplementation: New formulations, old questions. Haematologica.

[30] Gama L.A., Rocha Machado M.P., Beckmann A.P.S., Miranda J.R.d.A., Corá L.A., Américo M.F. (2020). Gastrointestinal motility and morphology in mice: Strain-dependent differences. Neurogastroenterol. Motil..

[31] Arike L., Seiman A., van der Post S., Rodriguez Piñeiro A.M., Ermund A., Schütte A., Bäckhed F., Johansson M.E.V., Hansson G.C. (2020). Protein Turnover in Epithelial Cells and Mucus along the Gastrointestinal Tract Is Coordinated by the Spatial Location and Microbiota. Cell Rep..

[32] Chassaing B., Aitken J.D., Malleshappa M., Vijay-Kumar M. (2014). Dextran Sulfate Sodium (DSS)-Induced Colitis in Mice. Curr. Protoc. Immunol..

[33] Bo T.B., Zhang X.Y., Kohl K.D., Wen J., Tian S.J., Wang D.H. (2020). Coprophagy prevention alters microbiome, metabolism, neurochemistry, and cognitive behavior in a small mammal. ISME J..

[34] Klindworth A., Pruesse E., Schweer T., Peplies J., Quast C., Horn M., Glöckner F.O. (2013). Evaluation of general 16S ribosomal RNA gene PCR primers for classical and next-generation sequencing-based diversity studies. Nucleic Acids Res..

[35] Martin M. (2011). Cutadapt removes adapter sequences from high-throughput sequencing reads. EMBnet. J..

[36] Callahan B.J., McMurdie P.J., Rosen M.J., Han A.W., Johnson A.J.A., Holmes S.P. (2016). DADA2: High-resolution sample inference from Illumina amplicon data. Nat. Methods.

[37] Callahan B. (2024). Silva Taxonomic Training Data Formatted for DADA2 (Silva Version 138.2).

[38] Altschul S.F., Gish W., Miller W., Myers E.W., Lipman D.J. (1990). Basic local alignment search tool. J. Mol. Biol..

[39] Sayers E.W., Bolton E.E., Brister J.R., Canese K., Chan J., Comeau D.C., Connor R., Funk K., Kelly C., Kim S. (2022). Database resources of the national center for biotechnology information. Nucleic Acids Res..

[40] McMurdie P.J., Holmes S. (2012). Phyloseq: A bioconductor package for handling and analysis of high-throughput phylogenetic sequence data. Pac. Symp. Biocomput..

[41] Cuisiniere T., Santos M.M. (2024). StackbarExtended: A user-friendly stacked bar-plot representation incorporating phylogenetic information and microbiota differential abundance analysis [version 1; peer review: 1 approved, 1 approved with reservations]. F1000Research.

[42] Love M.I., Huber W., Anders S. (2014). Moderated estimation of fold change and dispersion for RNA-seq data with DESeq2. Genome Biol..

[43] Caspi R., Altman T., Billington R., Dreher K., Foerster H., Fulcher C.A., Holland T.A., Keseler I.M., Kothari A., Kubo A. (2014). The MetaCyc database of metabolic pathways and enzymes and the BioCyc collection of Pathway/Genome Databases. Nucleic Acids Res..

[44] Douglas G.M., Maffei V.J., Zaneveld J.R., Yurgel S.N., Brown J.R., Taylor C.M., Huttenhower C., Langille M.G.I. (2020). PICRUSt2 for prediction of metagenome functions. Nat. Biotechnol..

[45] Wright R.J., Langille M.G.I. (2025). PICRUSt2-SC: An update to the reference database used for functional prediction within PICRUSt2. Bioinformatics.

[46] Ye J., Coulouris G., Zaretskaya I., Cutcutache I., Rozen S., Madden T.L. (2012). Primer-BLAST: A tool to design target-specific primers for polymerase chain reaction. BMC Bioinform..

[47] Stookey L.L. (1970). Ferrozine—A new spectrophotometric reagent for iron. Anal. Chem..

[48] Han J., Lin K., Sequeira C., Borchers C.H. (2015). An isotope-labeled chemical derivatization method for the quantitation of short-chain fatty acids in human feces by liquid chromatography-tandem mass spectrometry. Anal. Chim. Acta.

[49] Wells J.M. (2011). Immunomodulatory mechanisms of lactobacilli. Microb. Cell Fact..

[50] Dempsey E., Corr S.C. (2022). *Lactobacillus* spp. for Gastrointestinal Health: Current and Future Perspectives. Front. Immunol..

[51] Dera N., Kosińska-Kaczyńska K., Żeber-Lubecka N., Brawura-Biskupski-Samaha R., Massalska D., Szymusik I., Dera K., Ciebiera M. (2025). Impact of Early-Life Microbiota on Immune System Development and Allergic Disorders. Biomedicines.

[52] Roager H.M., Licht T.R. (2018). Microbial tryptophan catabolites in health and disease. Nat. Commun..

[53] de Groen P., Gouw S.C., Hanssen N.M.J., Nieuwdorp M., Rampanelli E. (2026). Early-Life Gut Microbiota: Education of the Immune System and Links to Autoimmune Diseases. Microorganisms.

[54] Huynh U., Zastrow M.L. (2023). Metallobiology of *Lactobacillaceae* in the gut microbiome. J. Inorg. Biochem..

[55] Zheng C., Zhong Y., Xie J., Wang Z., Zhang W., Pi Y., Zhang W., Liu L., Luo J., Xu W. (2023). *Bacteroides acidifaciens* and its derived extracellular vesicles improve DSS-induced colitis. Front. Microbiol..

[56] Seril D.N., Liao J., Ho K.L., Warsi A., Yang C.S., Yang G.Y. (2002). Dietary iron supplementation enhances DSS-induced colitis and associated colorectal carcinoma development in mice. Dig. Dis. Sci..

[57] Seril D.N., Liao J., Yang C.S., Yang G.Y. (2005). Systemic iron supplementation replenishes iron stores without enhancing colon carcinogenesis in murine models of ulcerative colitis: Comparison with iron-enriched diet. Dig. Dis. Sci..

[58] Mahalhal A., Burkitt M.D., Duckworth C.A., Hold G.L., Campbell B.J., Pritchard D.M., Probert C.S. (2021). Long-Term Iron Deficiency and Dietary Iron Excess Exacerbate Acute Dextran Sodium Sulphate-Induced Colitis and Are Associated with Significant Dysbiosis. Int. J. Mol. Sci..

[59] Medjbeur T., Sardo U., Perrier P., Cormier K., Roy M., Dumay A., Kautz L. (2025). Comparative analysis of dietary iron deprivation and supplementation in a murine model of colitis. FASEB BioAdv..

[60] Kronman M.P., Zaoutis T.E., Haynes K., Feng R., Coffin S.E. (2012). Antibiotic exposure and IBD development among children: A population-based cohort study. Pediatrics.

[61] Liu Y., Jiao C., Zhang T., Li X., Li P., Lu M., Ye Z., Du Y., Du R., Zhang W. (2023). Early-Life Gut Microbiota Governs Susceptibility to Colitis via Microbial-Derived Ether Lipids. Research.

[62] Ozkul C., Ruiz V.E., Battaglia T., Xu J., Roubaud-Baudron C., Cadwell K., Perez-Perez G.I., Blaser M.J. (2020). A single early-in-life antibiotic course increases susceptibility to DSS-induced colitis. Genome Med..

[63] Al Nabhani Z., Dulauroy S., Marques R., Cousu C., Al Bounny S., Déjardin F., Sparwasser T., Bérard M., Cerf-Bensussan N., Eberl G. (2019). A Weaning Reaction to Microbiota Is Required for Resistance to Immunopathologies in the Adult. Immunity.

[64] Gu K., Wu A., Yu B., Zhang T., Lai X., Chen J., Yan H., Zheng P., Luo Y., Luo J. (2023). Iron overload induces colitis by modulating ferroptosis and interfering gut microbiota in mice. Sci. Total Environ..

[65] Dogra S.K., Doré J., Damak S. (2020). Gut Microbiota Resilience: Definition, Link to Health and Strategies for Intervention. Front. Microbiol..

[66] Sprockett D., Fukami T., Relman D.A. (2018). Role of priority effects in the early-life assembly of the gut microbiota. Nat. Rev. Gastroenterol. Hepatol..

[67] McMillen S., Thomas S., Liang E., Nonnecke E.B., Slupsky C., Lönnerdal B. (2022). Gut Microbiome Alterations following Postnatal Iron Supplementation Depend on Iron Form and Persist into Adulthood. Nutrients.

[68] Hua R., Ding N., Hua Y., Wang X., Xu Y., Qiao X., Shi X., Bai T., Xiong Y., Zhuo X. (2025). Ligilactobacillus Murinus and Lactobacillus Johnsonii Suppress Macrophage Pyroptosis in Atherosclerosis through Butyrate-GPR109A-GSDMD Axis. Adv. Sci..

[69] Tang C., Kamiya T., Liu Y., Kadoki M., Kakuta S., Oshima K., Hattori M., Takeshita K., Kanai T., Saijo S. (2015). Inhibition of Dectin-1 Signaling Ameliorates Colitis by Inducing *Lactobacillus*-Mediated Regulatory T Cell Expansion in the Intestine. Cell Host Microbe.

[70] Okada T., Fukuda S., Hase K., Nishiumi S., Izumi Y., Yoshida M., Hagiwara T., Kawashima R., Yamazaki M., Oshio T. (2013). Microbiota-derived lactate accelerates colon epithelial cell turnover in starvation-refed mice. Nat. Commun..

[71] Hu J., Deng F., Zhao B., Lin Z., Sun Q., Yang X., Wu M., Qiu S., Chen Y., Yan Z. (2022). *Lactobacillus murinus* alleviate intestinal ischemia/reperfusion injury through promoting the release of interleukin-10 from M2 macrophages via Toll-like receptor 2 signaling. Microbiome.

[72] Alam A., Neish A. (2018). Role of gut microbiota in intestinal wound healing and barrier function. Tissue Barriers.

[73] Alam A., Leoni G., Quiros M., Wu H., Desai C., Nishio H., Jones R.M., Nusrat A., Neish A.S. (2016). The microenvironment of injured murine gut elicits a local pro-restitutive microbiota. Nat. Microbiol..

[74] Connors J., Dawe N., Van Limbergen J. (2018). The Role of Succinate in the Regulation of Intestinal Inflammation. Nutrients.

[75] Osaka T., Moriyama E., Arai S., Date Y., Yagi J., Kikuchi J., Tsuneda S. (2017). Meta-Analysis of Fecal Microbiota and Metabolites in Experimental Colitic Mice during the Inflammatory and Healing Phases. Nutrients.

[76] Westman E.L., McNally D.J., Rejzek M., Miller W.L., Kannathasan V.S., Preston A., Maskell D.J., Field R.A., Brisson J.R., Lam J.S. (2007). Identification and biochemical characterization of two novel UDP-2,3-diacetamido-2,3-dideoxy-alpha-D-glucuronic acid 2-epimerases from respiratory pathogens. Biochem. J..

[77] Dolan S.K., Wijaya A., Geddis S.M., Spring D.R., Silva-Rocha R., Welch M. (2018). Loving the poison: The methylcitrate cycle and bacterial pathogenesis. Microbiology.

[78] Nakamura A., Matsumoto M. (2025). Role of polyamines in intestinal mucosal barrier function. Semin. Immunopathol..

[79] Keller M.R., Kazi M.I., Saleh A., Basu U., Shin J.-H., Rhee K., Dörr T. (2026). Global response to antibiotic exposure reveals a critical role for nucleotide metabolism in high-level β-lactam tolerance. npj Antimicrob. Resist..

[80] Yilmaz B., Li H. (2018). Gut Microbiota and Iron: The Crucial Actors in Health and Disease. Pharmaceuticals.

[81] (2024). The BEGIN Study Bifidobacterium Infantis to Newborns: Effects of Modulating the Gut Microbial Composition on Growth, Immune Function and Inflammatory Conditions. ClinicalTrials.gov Identifier: NCT06452199. NCT06452199.

[82] Sandoval-Mosqueda I.L., Llorente-Bousquets A., Soto C., Márquez C.M., Fadda S., Del Río García J.C. (2023). *Ligilactobacillus murinus* Strains Isolated from Mice Intestinal Tract: Molecular Characterization and Antagonistic Activity against Food-Borne Pathogens. Microorganisms.

[83] Ryu H., Henson M., Elk M., Toledo-Hernandez C., Griffith J., Blackwood D., Noble R., Gourmelon M., Glassmeyer S., Santo Domingo Jorge W. (2013). Development of Quantitative PCR Assays Targeting the 16S rRNA Genes of *Enterococcus* spp. and Their Application to the Identification of Enterococcus Species in Environmental Samples. Appl. Environ. Microbiol..

[84] Badi S.A., Moradi H.R., Berimipour A., Shojaie S., Kariman A., Aval H.T., Seyedi S.A., Davari M., Sohouli M.H., Khatami S. (2025). Preventive and therapeutic effects of co-administration of *Bacteroides thetaiotaomicron* and infliximab on dextran sodium sulfate-induced colitis in mice. Intest. Res..

